# Faster but Not Truer, Part 1: A Systematic Scoping Review of Artificial Intelligence in Health Economics and Outcomes Research

**DOI:** 10.36469/001c.171937

**Published:** 2026-10-15

**Authors:** Onur Baser

**Affiliations:** 1 Graduate School of Public Health City University of New York, New York, USA

**Keywords:** artificial intelligence, machine learning, real-world evidence, health economics outcomes research, health technology assessment, causal inference

## Abstract

**Background:**

Artificial intelligence (AI) is being introduced into every stage of the health economics and outcomes research (HEOR) production line, from literature screening to causal estimation. The field lacks a defensible count of what has been done, and bibliometric claims about AI adoption circulate without measured error rates.

**Methods:**

Following PRISMA-ScR, we searched PubMed for AI terms co-occurring with HEOR terms for publications from 2010 through 2025, retrieved 2642 records, and included 2476 after screening. Records were classified into 8 nonexclusive application domains and 1 of 6 method categories using a published term dictionary. To quantify classification error, an independent large language model classifier, blinded to the dictionary labels and given only title and abstract, reclassified a random sample of 300 included records. We report per label counts, agreement, Cohen kappa, prevalence-adjusted bias-adjusted kappa, precision, recall, F1, and a precision-adjusted corpus size with exact binomial intervals. We separately assembled the primary performance evidence for each HEOR workflow, requiring a quantitative metric from a primary study.

**Results:**

Included publications rose from 29 in 2010 to 852 in 2025, a factor of 29.4, with the inflection at 2018 to 2019 rather than at the release of ChatGPT. AI reached 6.0% of all indexed HEOR output in 2025 while HEOR fell to 0.86% of all indexed medical AI output. Economic evaluation was the most frequently assigned domain (1559/2476, 63.0%), followed by real-world data and causal inference (496, 20.0%) and governance, reporting, and validity (494, 20.0%). Generative AI appeared in no record before 2021 and in 103 in 2025. Only 154 of 300 sampled records (51.3%; 95% confidence interval [CI], 45.7-56.9) were judged genuinely HEOR relevant, implying a precision-adjusted corpus of 1271 studies (95% CI 1131-1410). Agreement was substantial for real-world data and causal inference (kappa, 0.729) but negligible for economic evaluation (kappa, 0.144), where dictionary precision was 29.6%. Of 2476 records, 38 (1.5%) were randomized trials and 17 (0.7%) validation studies. The replication package is publicly available.

**Conclusions:**

The literature is growing faster than its own measurement infrastructure. Measured gains concentrate in tasks with observable ground truth, and predictive performance repeatedly fails to predict evidentiary performance.

## INTRODUCTION

Two facts about artificial intelligence (AI) in health economics and outcomes research (HEOR) sit uneasily beside each other. This series is about the distance between them.

The first is that the field has been reorganized around it. Since 2016, the US Food and Drug Administration (FDA) has received more than 500 drug and biological product submissions containing AI components.^1–3^ The agency issued its first draft guidance on AI for regulatory decision making in January 2025,^4^ joined the European Medicines Agency in publishing 10 guiding principles for good AI practice in drug development in January 2026,^5,6^ and has now authorized 1614 AI enabled medical devices.^7^ The International Society for Pharmacoeconomics and Outcomes Research moved AI from the third-ranked HEOR trend for 2024 to 2025 to the first-ranked trend for 2026 to 2027,^8^ and published a taxonomy, a reporting guideline, and a policy report for generative AI in HEOR within a single year.^9–11^

The second is that almost none of it has reached the documents on which reimbursement decisions are made. In a systematic audit of 1391 health technology assessment (HTA) documents submitted to 3 agencies between 2023 and 2025, exactly 3 disclosed the use of AI or machine learning, an adoption rate of 0.2%.^12^ When editors of *The Lancet* checked every submission received during February 2024, 0.6% declared use of ChatGPT.^13^

That gap is a measurement problem before it is anything else, and the measurement problem is the subject of Part 1. We do not have a defensible count of what has actually been done. Prior reviews have been deliberately partial. Lee et al screened 148 records for wearable applications and included 32,^14^ then sampled 161 of 805 records for nonwearable applications and included 92.^15^ A 2026 scoping review of the economic aspects of machine learning in health care screened 6332 references and included 18,^16^ and a protocol for a review of machine learning in health economic evaluation screened 4141 records and found 30 eligible reports.^17^ Each answers a narrower question than the one practitioners now ask, which is how large the field is, what it is actually doing, and how fast its composition is changing.

The reason the answer matters beyond bibliometrics is that the standards now being written for AI in evidence generation are being written against an evidence base whose reliability nobody has quantified. All the current frameworks are risk based, and all of them presume the submitter can characterize the risk. Yet the prediction model literature they inherit is dominated by high risk of bias: 545 of 606 COVID-19 prediction models were rated at high risk of bias,^18^ as were 148 of 171 studies of machine learning–based prediction models.^19^

This article therefore does three things. First, it reports an original systematic scoping review of the indexed literature following PRISMA-ScR, covering 2010 through 2025, with a full and reproducible search rather than a sample. Second, and unusually for a scoping review, it validates its own classification against an independent blinded classifier and reports the resulting error rates, because a bibliometric claim without a measured error rate is an assertion. Third, it sets the measured performance evidence for each HEOR workflow beside that map, so that the size and the quality of a literature can be read together. Part 2 takes up the regulatory, health technology assessment, and industry record, the economics of automation as it applies to this field, and a proposed reporting standard. The intended readers are the people who will have to defend this evidence: HEOR scientists and biostatisticians in industry and academia, HTA methodologists writing the next generation of position statements, and reviewers who will receive dossiers built with these tools whether the dossiers say so.

## METHODS

We followed the Preferred Reporting Items for Systematic Reviews and Meta-Analyses extension for Scoping Reviews (PRISMA-ScR). The protocol, all search strings, the classification dictionary, and the analysis code are archived in a public replication package.^20^ No patient level data were used, and no ethics approval was required.

### Search Strategy and Eligibility

We searched PubMed on 9 September 2026 using a structured Boolean query requiring co-occurrence of at least 1 AI term and at least 1 HEOR term in the title, abstract, or MeSH terms, restricted to publication years 2010 through 2025. AI terms covered a*rtificial intelligence, machine learning, deep learning, neural networks, natural language processing, large language models, generative AI,* and *named model families.* HEOR terms covered *cost effectiveness, cost utility, economic evaluation, decision analytic modeling, budget impact, health technology assessment, real world evidence, comparative effectiveness, patient reported outcomes, utilities, quality adjusted life years,* and *health care resource utilization.* The complete query strings are reproduced in **Supplementary Table S1**.

Records were eligible if they were journal articles, reviews, or conference proceedings published in English between 2010 and 2025 with a retrievable abstract. We excluded editorials, letters, comments, preprints, retracted publications, and records dated outside the window, and removed duplicates on PubMed identifier and normalized title.

To place the corpus in context, we retrieved annual denominator series from PubMed using the same interface, reported in **Supplementary Table S5**. One discrepancy should be noted. The range form of the query returned 2656 records where the sum of the 16 per-year queries was 2929, a difference of 273, most likely from MedlineDate records counted in 2 calendar years. We used the per-year series for trends and the range query for the PRISMA flow.

### Classification

Each included record was classified by a deterministic term dictionary applied to the concatenated title, abstract, and MeSH terms. The dictionary assigns 8 nonexclusive application domains: economic evaluation; decision modeling and simulation; evidence synthesis; real-world data and causal inference; outcome and cost prediction; patient-reported outcomes and preferences; HTA, payer, and market access; and governance, reporting, and validity. It separately assigns a single most specific method category from six: generative AI and large language models; natural language processing before the generative era; deep learning; classical machine learning; artificial intelligence–unspecified; and no AI method term identified. Domain labels are nonexclusive because a single paper frequently reports, for example, a natural language processing pipeline used to construct a cohort for a cost outcome. The full dictionary is provided in **Supplementary Table S2**.

### Validation of the Classifier

A dictionary is fast, transparent, and reproducible, and it is also wrong in ways that are invisible unless measured. We drew a simple random sample of 300 of the 2476 included records and had an independent large language model classifier, blinded to the dictionary output and given only title and abstract, answer 2 questions: whether the record was genuinely relevant to HEOR as a practitioner would define it, and which of the same 8 domains and 6 method categories applied. Prompts and raw outputs were archived.

We reported, for every label, the number assigned by each classifier, observed agreement, Cohen kappa, prevalence-adjusted bias-adjusted kappa (PABAK), and the precision, recall, and F1 of the dictionary treating the blinded classifier as the reference. PABAK was reported alongside kappa because kappa is unstable at extreme prevalence and several labels are rare. We also reported search precision, defined as the proportion of sampled records judged genuinely HEOR relevant, with an exact binomial 95% confidence interval (CI), and a precision-adjusted corpus size obtained by applying that proportion and its interval to the full included count. We treated the blinded classifier as a reference standard, not as truth, and returned to that choice in the Limitations.

### Narrative Evidence Review

Alongside the bibliometric review we assembled a source grounded evidence base on the measured performance of AI in each HEOR workflow, requiring a primary study reporting a quantitative metric. Where a figure could not be confirmed from a retrieved page, we recorded it as not available rather than estimating it. Gray literature without a digital object identifier was cited with the issuing organization, date, and uniform resource locator.

### Statistical Analysis

Counts were reported with percentages of the relevant denominator and CIs for proportions are exact binomial (Clopper-Pearson). Growth was expressed as a ratio of endpoint counts. No hypothesis tests were reported for the bibliometric series because the series are complete counts rather than samples. Analyses used Python 3.11, with versions pinned in the replication package.

## RESULTS

### Size and Shape of the Literature

The search identified 2642 records. After removal of 14 duplicates, 2628 were screened and 152 excluded: 70 without a retrievable abstract, 38 not in English, 19 editorials, 10 preprints, 7 letters, 4 retracted, and 4 with a publication year outside the window. This left 2476 included records. **Figure 1** presents the PRISMA-ScR flow, itemized in **Supplementary Table S3,** and **Table 1** summarizes the corpus.

**Figure 1.**
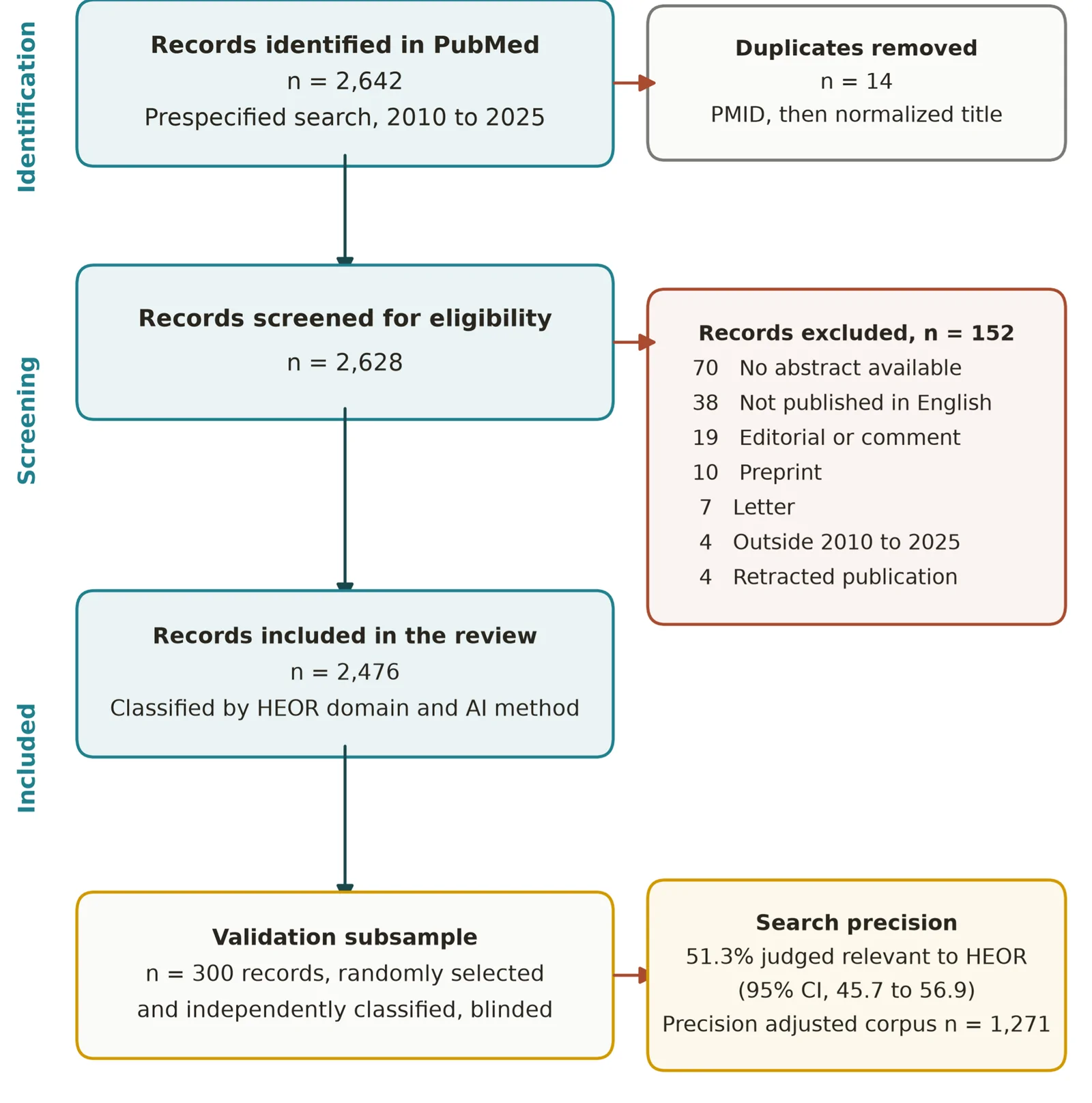
PRISMA-ScR Flow of Study Identification, Screening, and Inclusion Records identified in PubMed for publication years 2010 through 2025, with duplicate removal, itemized exclusions, and the 2476 included records. The precision-adjusted corpus estimate derived from blinded independent classification of 300 randomly sampled included records is shown for comparison.

**Table 1.** Characteristics of the Included Corpus (N = 2476)

**Characteristic**	**Included Records (N)**	**% of Included**	**Classification Reliability**
Publication year			–
2010	29	1.17	–
2011	28	1.13	–
2012	47	1.90	–
2013	31	1.25	–
2014	34	1.37	–
2015	24	0.97	–
2016	23	0.93	–
2017	36	1.45	–
2018	48	1.94	–
2019	91	3.68	–
2020	130	5.25	–
2021	163	6.58	–
2022	232	9.37	–
2023	270	10.90	–
2024	438	17.69	–
2025	852	34.41	–
Application domain (nonexclusive)			Cohen kappa vs blinded classifier
Economic evaluation	1559	62.96	0.144
Real-world data and causal inference	496	20.03	0.729
Governance, reporting, and validity	494	19.95	0.546
Outcome and cost prediction	393	15.87	0.453
Evidence synthesis	283	11.43	0.666
HTA, payer, and market access	209	8.44	0.576
Decision modeling and simulation	115	4.64	0.579
Patient-reported outcomes and preferences	76	3.07	0.306
No domain term identified	278	11.23	–
Method category (most specific, exclusive)			
Artificial intelligence–unspecified	682	27.54	0.667 (6-category multiclass)
Classical machine learning	615	24.84	–
Deep learning	445	17.97	–
No AI method term identified	445	17.97	–
Generative AI and large language models	159	6.42	–
Natural language processing (pre-generative)	130	5.25	–
Leading journals			
*Scientific Reports*	79	3.19	–
*Sensors (Basel, Switzerland)*	60	2.42	–
*Cureus*	43	1.74	–
*Journal of Medical Internet Research*	41	1.66	–
*BMJ Open*	36	1.45	–
*PloS One*	35	1.41	–
*Value in Health*	32	1.29	–
*Journal of Clinical Medicine*	27	1.09	–
*NPJ Digital Medicine*	26	1.05	–
*Frontiers in Public Health*	20	0.81	–
Indexed publication type			
Journal article	2471	99.80	–
Review	674	27.22	–
Research support, non-US government	492	19.87	–
Systematic review	121	4.89	–
Research support, NIH, extramural	105	4.24	–
Comparative study	91	3.68	–
Multicenter study	54	2.18	–
Scoping review	42	1.70	–
Randomized controlled trial	38	1.53	–
Research support, US government, non-PHS	37	1.49	–
Clinical trial protocol	31	1.25	–
Meta-analysis	27	1.09	–

Annual output rose from 29 records in 2010 to 852 in 2025, a factor of 29.4, with the inflection at 2018 to 2019 rather than at the release of ChatGPT. The series was 29, 28, 47, 31, 34, 24, 23, 36, 48, 91, 130, 163, 232, 270, 438, and 852, the largest proportional increase being the rise of 89.6% from 2018 to 2019. **Figure 2** shows the series against its denominators and **Supplementary Table S4** gives counts per year by domain.

**Figure 2.**
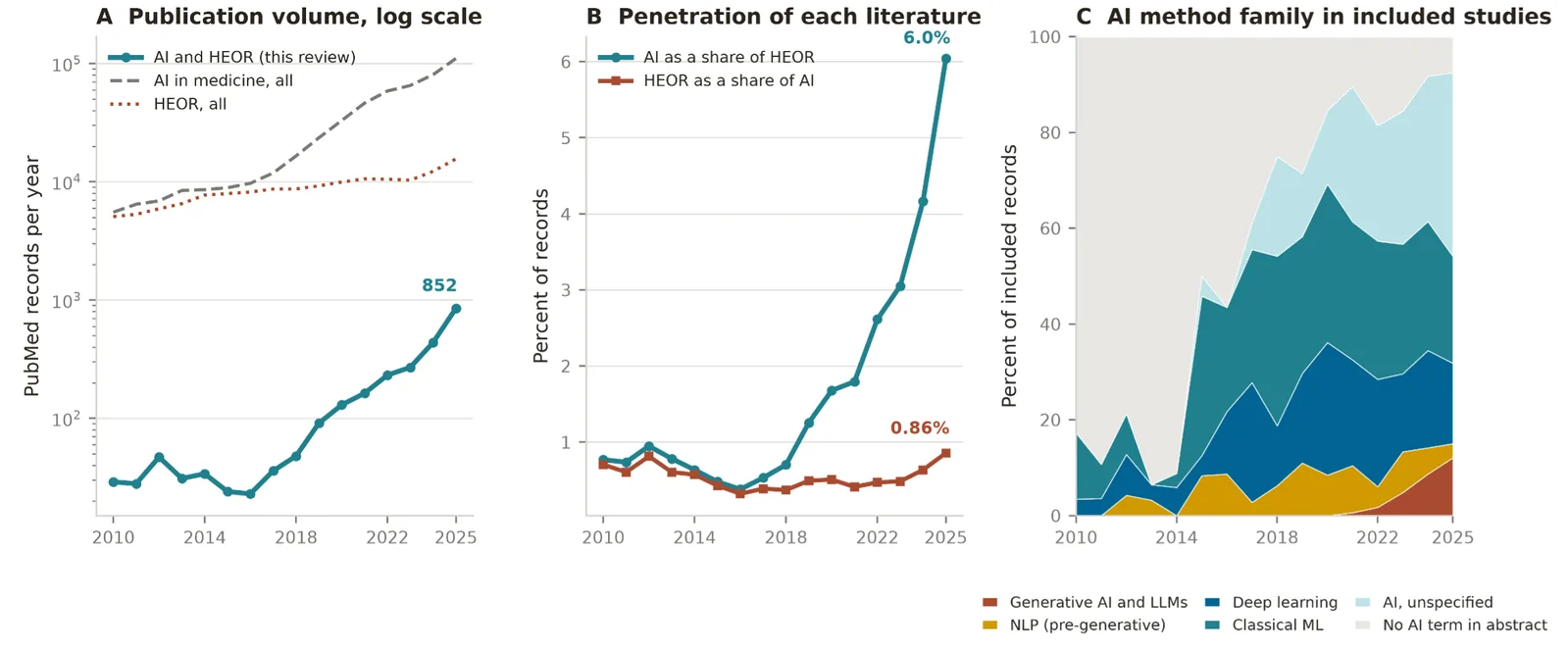
Growth and Composition of the AI and HEOR Literature, 2010-2025, Against Its Denominators **A**: Publication volume on a logarithmic scale for the included corpus, for all indexed medical AI publications, and for all indexed HEOR publications. **B**: Penetration of each literature into the other (ie, AI-containing HEOR publications as a percentage of all indexed HEOR publications, which reached 6.0% in 2025, and HEOR publications as a percentage of all indexed medical AI publications, which reached 0.86%). **C**: The method family composition of the included corpus as a percentage of records per year, with generative AI and large language models, pregenerative natural language processing, deep learning, classical machine learning, artificial intelligence–unspecified, and records with no AI method term in the abstract.

Two ratios matter more than the raw count. AI containing publications reached 6.0% of all indexed HEOR output in 2025, having been 0.8% in 2010, while HEOR fell to 0.86% of all indexed medical AI output, since PubMed indexed 110 494 AI in medicine records against 15 641 HEOR records that year. That asymmetry means HEOR will be a consumer of externally developed tools far more often than a producer of them, inheriting validation practices set elsewhere.

Domain composition is reported in **Table 1** and mapped in **Figure 3**. Economic evaluation was the most frequently assigned domain, appearing in 1559 of 2476 records (63.0%), followed by real-world data and causal inference (496, 20.0%), governance, reporting, and validity (494, 20.0%), outcome and cost prediction (393, 15.9%), evidence synthesis (283, 11.4%), HTA, payer, and market access (209, 8.4%), decision modeling and simulation (115, 4.6%), and patient-reported outcomes and preferences (76, 3.1%). No domain term was assigned in 278 records (11.2%). Method composition was artificial intelligence–unspecified in 682 records (27.5%), classical machine learning in 615 (24.8%), deep learning in 445 (18.0%), no identifiable AI method term in 445 (18.0%), generative AI or large language models in 159 (6.4%), and pre-generative natural language processing in 130 (5.3%).

**Figure 3.**
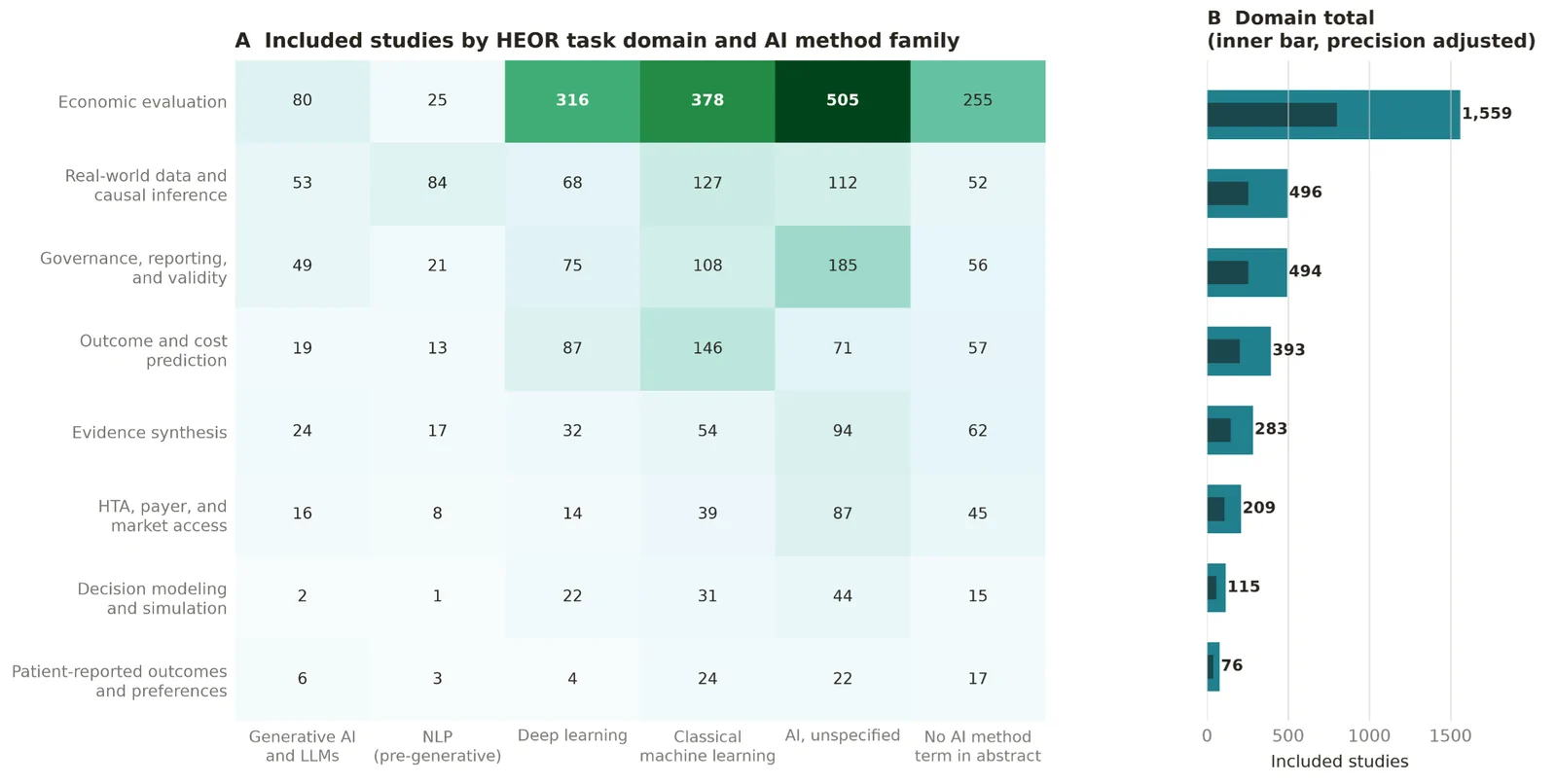
Evidence Map of AI Applications Across HEOR Tasks **A**: Cross-tabulation of the 8 application domains against the 8 method families; cell values are counts of included records and domain labels are nonexclusive, so rows do not sum to the corpus. **B**: The domain total for each row, with the precision-adjusted total shown as an inner bar so that the measured and the nominal size of each literature can be read together.

The generative series is empty before 2021, then 1, 4, 13, 38, and 103 records for 2021 through 2025. Growth is steep and the level is low: even in 2025 generative AI appeared in 12.1% of output. The fastest growing domain label in absolute terms was governance, reporting, and validity, which rose from 82 records in 2024 to 237 in 2025. The field is now writing more about how to govern these tools than it is publishing indexed applications of them.

Venues were highly dispersed across more than 900 distinct journals, led by *Scientific Reports* (79 records, 3.2%), *Sensors* (60, 2.4%), *Cureus* (43, 1.7%), *Journal of Medical Internet Research* (41, 1.7%), and *Value in Health* (32, 1.3%). That the field’s own specialty journal accounts for 1.3% of the corpus is the important observation: the literature HEOR practitioners will be asked to appraise is overwhelmingly published outside HEOR venues and therefore reviewed by people who do not routinely apply HEOR reporting standards. **Supplementary Table S6** lists the 40 most frequent journals.

Study designs were correspondingly weak on the dimensions HTA reviewers care about. Of 2476 records, 674 (27.2%) were indexed as reviews and 121 (4.9%) as systematic reviews, against 38 (1.5%) randomized controlled trials and 17 (0.7%) validation studies. **Supplementary Table S7** gives the full distribution.

### What Validation Reveals

The blinded independent classification of 300 randomly sampled records produced the most consequential result in this review, and it is not a flattering one.

Only 154 of 300 sampled records (51.3%; 95% CI, 45.7-56.9) were judged genuinely relevant to health economics and outcomes research. Applying that proportion to the corpus gives a precision-adjusted estimate of 1271 genuinely HEOR relevant studies (95% CI, 1131-1410) rather than 2476. Precision was 52.4% (95% CI, 46.5-58.3) among the 271 sampled records to which the dictionary had assigned at least 1 domain and 41.4% (95% CI, 25.5-59.3) among the 29 to which it had assigned none, so the dictionary carries signal, but not nearly enough of it. **Table 2** and **Figure 4** report the validation in full, with the stratified precision in **Supplementary Table S9**.

**Table 2.** Validation of the Classification Against Blinded Independent Classification of 300 Randomly Sampled Records

**Label**	**Assigned by Dictionary (n)**	**Assigned by Blinded Classifier (n)**	**Observed Agreement (%)**	**Cohen Kappa**	**PABAK**	**Precision (%)**	**Recall (%)**	**F1 (%)**
Economic evaluation	186	69	51.7	0.144	0.033	29.6	79.7	43.1
Decision modeling and simulation	16	30	94.0	0.579	0.880	87.5	46.7	60.9
Evidence synthesis	33	46	92.3	0.666	0.847	84.8	60.9	70.9
Real-world data and causal inference	64	75	90.3	0.729	0.807	85.9	73.3	79.1
Outcome and cost prediction	43	50	85.7	0.453	0.713	58.1	50.0	53.8
Patient-reported outcomes and preferences	8	22	93.3	0.306	0.867	62.5	22.7	33.3
HTA, payer, and market access	21	25	94.0	0.576	0.880	66.7	56.0	60.9
Governance, reporting, and validity	65	68	84.3	0.546	0.687	66.2	63.2	64.7
Method category (6-category multiclass)	300	300	73.7	0.667				
Generative AI or LLM flag			97.0	0.674	0.940			
								
**Search Precision**	**Sampled (n)**	**Judged HEOR-Relevant (n)**	**Precision (%)**	**95% CI Lower**	**95% CI Upper**			
All included records	300	154	51.3	45.7	56.9			
Dictionary assigned ≥1 HEOR domain	271	142	52.4	46.5	58.3			
Dictionary assigned no HEOR domain	29	12	41.4	25.5	59.3			
Precision-adjusted corpus size	2476 included	1271		1131	1410			

Per-label counts, observed agreement, Cohen kappa, PABAK, precision, recall, and F1, with search precision and the precision-adjusted corpus estimate.

**Figure 4.**
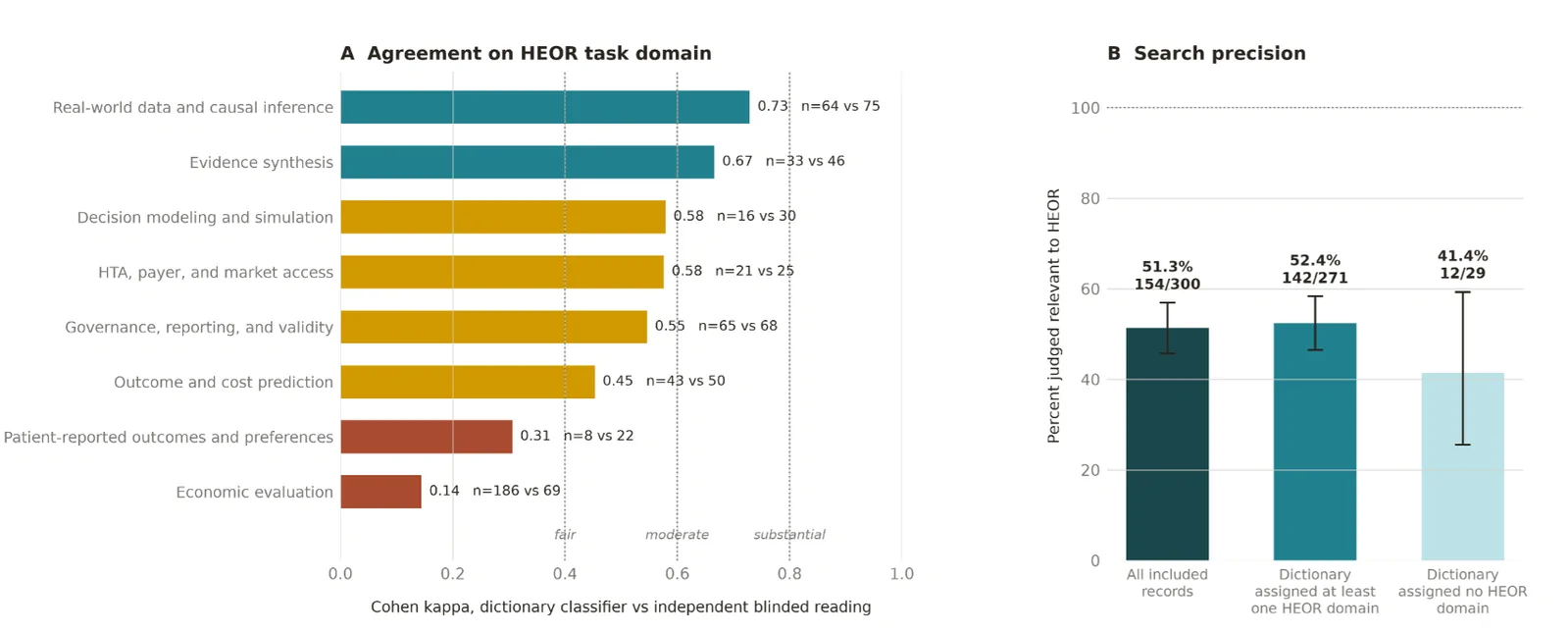
Validation of the Term Dictionary Against Blinded Independent Classification of 300 Randomly Sampled Included Records **A:** Cohen kappa for agreement on each HEOR task domain, annotated with the number of records the dictionary and the blinded classifier each assigned to that domain, and shaded by the conventional interpretive bands for fair, moderate, and substantial agreement. **B:** Search precision overall and stratified by whether the dictionary assigned at least 1 domain label or none, with exact binomial 95% CIs. Full agreement statistics, including prevalence-adjusted bias-adjusted kappa, precision, recall, and F1, are given in **Table 2** and **Supplementary Table S8**.

Per-label agreement was heterogeneous in a way that maps almost exactly onto how well specified each concept is. Agreement was substantial for real-world data and causal inference (Cohen kappa, 0.729; observed agreement, 90.3%; F1, 79.1%) and moderate to substantial for evidence synthesis (kappa, 0.666; F1, 70.9%), decision modeling and simulation (kappa, 0.579), HTA, payer, and market access (kappa, 0.576), and governance, reporting, and validity (kappa, 0.546). It was fair for outcome and cost prediction (kappa, 0.453) and poor for patient-reported outcomes and preferences (kappa, 0.306, F1, 33.3%). Method classification agreed at kappa 0.667 across 6 categories and the generative AI flag at kappa 0.674. Full statistics are in **Supplementary Table S8**.

The exception should change how bibliometric claims about this field are read. For economic evaluation, the largest and fastest growing label in the corpus, Cohen kappa was 0.144 with observed agreement of 51.7% and PABAK of 0.033. The dictionary assigned this label to 186 of 300 sampled records; the blinded classifier assigned it to 69. Dictionary precision was 29.6% with recall 79.7%. In plain terms, the dictionary treats any paper that mentions cost, resource use, or efficiency as an economic evaluation, and roughly 7 in 10 of those are not economic evaluations in the sense a reimbursement reviewer would use.

Two implications follow. First, our own headline count of 1559 economic evaluation records is an upper bound with an implied true value near 574 by the precision ratio, and any bibliometric claim about the growth of AI in health economic evaluation that does not report a measured classification error rate should be treated the same way. Second, the ranking of kappa values is a diagnostic of conceptual clarity in the field itself: real-world data and causal inference has a shared vocabulary and scores well, patient-reported outcomes and preferences does not and scores worst.

### Evidence Synthesis: The Best Measured Workflow

Evidence synthesis is where the performance evidence is strongest and where it is most consistently misread. **Supplementary Table S10** lists the screening studies. The best result in the literature is Matsui et al, who applied GPT-4 to 1381 and 3146 record sets and reported adjusted sensitivity of 0.962 and specificity of 0.996 at about 110 records per minute and a cost of US$59.^21^ It has not been reproduced at scale independently. Tran et al, on 22 665 citations, reported sensitivity between 81.1% and 96.5% but specificity between 25.8% and 80.4%, generating 10,279 false positives in the worst configuration.^22^ Oami et al, on 16 669 citations, reported sensitivity of 0.85 and specificity of 0.98 but a median specificity gap of 0.48 across reviews.^23^ Khraisha et al found GPT-4 sensitivity of 0.42 with kappa 0.34 against human reviewer kappa of 0.77.^24,25^

Read together, these say something actionable: sensitivity is acceptable, specificity is unstable across topics, and the variance between reviews exceeds the difference between models. That is the profile of a second screener with human adjudication, not of a sole screener. It is the conclusion of the joint position statement of Cochrane, the Campbell Collaboration, JBI, and the Collaboration for Environmental Evidence,^26^ and why the ISPOR agentic framework reports screening F1 between 0.917 and 0.977 under a human in the loop design rather than an autonomous one.^27^

Data extraction performs better than screening, and **Supplementary Table S11** gives the studies. Gartlehner et al extracted 160 items with Claude 2 at 96.3% accuracy,^28^ and Liu et al reported 94.8% accuracy at 88 seconds per randomized trial.^29^ The most informative study ran 12 814 extractions across 107 randomized trials and found Claude 3.5 at 96.2% accuracy against human extractors at 95.3% and human extraction assisted by the model at 97.9%, with extraction time falling from 86.9 minutes to 14.7.^30^ The point is not that the model beat the humans, since the difference sits inside plausible noise. The point is that the combination beat both.

Downstream analytic steps automate as well. Reason et al had GPT-4 write 30 R scripts to replicate published cost effectiveness models; in the non-small cell lung cancer set 14 of 15 scripts were error free, and every error free script reproduced the published incremental cost effectiveness ratio to within 1%.^31,32^ A systematic review of generative AI in health economic evaluation screened 2322 records and included 25 studies, of which 92% were conference abstracts and 96% were company authored.^33^ That composition is the finding.

The economic case rests on a smaller base than the enthusiasm suggests. Abogunrin et al found 17 of 25 studies reporting time reductions above 50%, against a per review cost above US $141 000.^34,35^ The most honest comparison available is the ISPOR time and motion study of 234 publications: a hybrid human and AI workflow achieved 97.0% accuracy with 80.6% recall and 51.4% time savings, a fully automated workflow achieved 81.1% time savings with recall of only 48.4%, and unaided humans achieved 90.1% accuracy with 88.7% recall.^27^ A workflow that misses half the eligible evidence is not 81% cheaper. It is a different study.

### Real-World Data and Causal Inference

This is the domain our validation ranked most coherent, and the domain where the evidence most directly contradicts the intuition that better prediction yields better inference. **Supplementary Table S12** maps the methods.

The foundational result is Schneeweiss et al on high-dimensional propensity scores, where the estimated relative risk in a cohort of 49 653 users moved from 1.09 crude to 0.94 with 15 investigator specified covariates and to 0.88 with 500 algorithmically selected covariates.^36–38^ Benasseur et al showed the failure mode plainly: high-dimensional propensity score weighting produced bias of 0.094 with 23.6% CI coverage where a modified procedure achieved bias of 0.018 with 91.4% coverage.^39^ Coverage of 23.6% for a nominal 95% interval is not a subtle problem.

The machinery is mature. Targeted maximum likelihood estimation,^40^ super learning,^41^ causal forests,^42^ and double or debiased machine learning^43^ are in routine use, and their translation into HEOR has been mapped through causal forests for heterogeneity in cost effectiveness analysis,^44,45^ integration of decision modeling with machine learning based causal estimation,^46^ a scoping review yielding 74 studies of heterogeneous treatment effect estimation with real-world data,^47^ and transportability methods.^48–50^

What the field lacks is calibration of expectations, and the clearest demonstration is a trial emulation. Wang et al emulated PARADIGM-HF in Veterans Affairs data, where the randomized hazard ratio was 0.81 (95% CI, 0.61-1.06). A logistic propensity score model with area under the curve of 0.79 yielded 0.93; a gradient-boosted model with area under the curve of 0.95 yielded 0.61.^51^ The model with the best discrimination produced the estimate furthest from the trial benchmark. Propensity score models are not prediction problems, and optimizing separation of treated from untreated can degrade the covariate balance that identification requires. It is, however, exactly the inference a reviewer would draw from an area under the curve reported without a balance diagnostic, and it is why FDA guidance on real-world data declines to endorse any specific AI technology,^52^ why the target trial framework has been recommended as the organizing discipline,^53^ and why the PALISADE checklist was written.^54^

### Unstructured Data and AI-Derived Endpoints

Approximately 80% of clinical data is unstructured, and this is the strongest affirmative case for AI in HEOR.^55^ The gains are not marginal. Social and behavioral determinants recorded in claims at prevalences between 0.03% and 8.3% were recorded at between 1.40% and 36.3% once notes were processed, with lack of social support identifiable only from text in 99.8% of cases.^56^ In an oncology cohort, 44 of 48 patients (91.7%) with adverse social determinants documented in text had no corresponding Z code.^57^

Performance on specific extraction tasks is good and uneven, as **Supplementary Table S13** shows. Xu et al, across 79 698 patients and 495 862 notes, an achieved area under the receiver operating characteristic curve of 0.95 for performance status and found that imputed poor performance status carried a hazard ratio of 11.9.^58^ A Veterans Affairs implementation reported 98.5% accuracy^59^ and programmed death ligand 1 extraction reached micro F1 of 0.925,^60^ while one commercial oncology model reported precision of 98.8% for stage value but recall of only 79% for linking histology to neoplasm,^61^ and in another registry task T stage extraction reached F1 of 0.89 while M stage reached 0.24.^62^ The pattern is that the variable a cohort definition depends on is often the variable extracted worst.

Two results show why this matters for an estimate rather than for a metric. Varma et al derived real-world progression-free survival two ways in the same cohort and obtained 30 months from radiology reports against 23 months from clinical notes, with median real-world progression-free survival of 20 months (95% CI, 18-25) against 25 months (95% CI, 15-35) by manual curation in a validation subset.^63^ A 7-month difference in a median attributable to which document type was read is a difference that changes a cost effectiveness result. Separately, adding natural language processing features raised propensity score model area under the curve from 0.66 to 0.77 and from 0.80 to 0.89 while the estimated hazard ratios barely moved, which is the PARADIGM-HF lesson in another guise.

Vendor validation frameworks have begun to respond. Flatiron Health published the VALID framework, requiring variable-level benchmarking against expert human abstraction, internal consistency checks, replication, and subgroup stratified bias assessment,^64,65^ and its machine learning extracted real-world response correlated with human abstraction at *r* = 0.86 in a test subset of 4047.^66–68^ Verantos and coauthors from Amgen, Johnson and Johnson, and GSK reported recall improving from 49.9% to 93.2% and completeness from 46.1% (95% CI, 38.2-54.0) to 96.6% (95% CI, 85.8-107.4).^69^ A reported CI with an upper bound of 107.4% for a proportion is itself a reporting problem, and all of these validation efforts were authored by parties with a commercial interest in the result.^70^

The decisive gap is regulatory. FDA has accepted electronic health record–derived real-world data in a label expansion, approving palbociclib for men with advanced breast cancer on the basis of records from three databases.^71^ That establishes acceptance of an electronic health record derived source, not acceptance of an AI derived endpoint, and we were unable to document a single instance of any regulator or HTA body explicitly accepting or rejecting one as such. For a sponsor deciding whether to build a submission on real-world progression, that absence is the single most important fact in this series.

### Decision Modeling, Simulation, and Synthetic Data

Decision modeling is the smallest domain (115 records, 4.6%) and contains the cleanest efficiency results, because its tasks are computational rather than inferential. Metamodeling reduced an expected value of partial perfect information calculation requiring 12.5 million model runs from more than 1000 days of brute force computation to about two days,^72^ and automated model coding replicated published incremental cost effectiveness ratios to within 1%.^11,31,73–75^

Decision modeling is also the part of HEOR where uncertainty is already formalized, and therefore the part where AI is most easily disciplined. Otten et al cataloged 80 methods for assessing uncertainty in decision analytic models, nearly all of which presuppose that the uncertainty has been quantified,^76,77^ and that presupposition is what an opaque model breaks. Value of information analysis^78–81^ and the CHEERS 2022 requirement that inputs and their sources be reported^82^ define what an AI-assisted model must still deliver.

Synthetic data is where the promise is largest and the reporting weakest, and **Supplementary Table S14** collects the studies. The utility evidence is genuine: D’Amico et al generated synthetic data for 7133 patients with clinical fidelity of 93% and genomic fidelity of 90% and constructed a synthetic control arm of 187 matched patients,^83,84^ and Wang et al built AI-generated digital twins from 6736 subjects in Alzheimer disease, in which 6236 of 6728 subjects with recorded race were White and 152 were Black,^85^ a composition that determines what the resulting population can be used to claim.

Generator choice dominates the result. Akiya et al found that classification and regression tree–based generation produced synthetic data sets whose median progression-free survival fell inside the real 95% CI in 88.8% to 98.1% of replicates, against 6.5% to 37.8% for a conditional tabular generative adversarial network.^86^ A generator that recovers the target quantity in 6.5% of replicates is not a lower quality version of one that recovers it in 98%; it is unusable for that purpose, and nothing in the summary statistics of the synthetic data would tell you which one you had. Reporting has not kept pace: of 73 studies, 70 (95%) evaluated utility while only 31 of 67 (46%) claiming a privacy purpose evaluated privacy, and only 8 of 73 (11%) used a differentially private generator.^87^

## DISCUSSION

Two findings organize this first article, and both are about measurement. The first is that the size of this literature cannot be read from a keyword count, which overstates it by a factor of two. A corpus of 2476 records reduces to approximately 1271 genuinely HEOR-relevant studies (95% CI, 1131-1410) under blinded independent classification, and the label that grew fastest, economic evaluation, is the label our own classifier got most wrong (Cohen kappa, 0.144, precision 29.6%). A count without an error rate is a marketing figure, and if a dictionary built specifically for this task and published in full achieves precision of 29.6% on the single most important label, then keyword-based counts circulating in conference abstracts and market reports should be read as upper bounds of unknown looseness.

The second is a methodological finding that recurs in three independent places and should be stated as a rule. In the PARADIGM-HF emulation, a propensity model with area under the curve of 0.95 produced a hazard ratio of 0.61 against a trial value of 0.81, while a weaker model at 0.79 produced 0.93.^51^ In the natural language processing–augmented propensity work, discrimination rose substantially while the effect estimates barely moved. In drift monitoring across 4 deployed systems, calibration degraded before discrimination did.^88^ The rule is that predictive performance is not evidentiary performance: HEOR quantities are calibrated, counterfactual, and decision relevant, and none of those properties is measured by a receiver operating characteristic curve. A dossier that reports model performance without reporting the sensitivity of the decision to that model has reported the wrong thing.

The two findings share a structure. Where a task has an observable answer, the gains are real, replicated, and often large: extraction accuracy against human abstraction at 96.2% against 95.3%,^30^ screening recall against a human reference,^27^ code generation against a published incremental cost effectiveness ratio,^31^ and computation against brute force.^72^ Where the answer is not observable, there is no reported instance of AI improving the accuracy of a cost effectiveness estimate, because accuracy of a cost effectiveness estimate is not measurable. The kappa ranking in our own validation reproduces the same division from the outside.

The consequence for practice is that the two layers should be governed differently. Clerical automation needs throughput and audit trails. Inferential use needs a decision-level sensitivity analysis, because nothing else in the toolkit will detect the failure. Part 2 takes up what regulators and HTA agencies have actually said about this division, what industry reports, and what a reporting standard built for it would require.

### Limitations

We searched a single database. PubMed does not index much of the conference and gray literature where AI in HEOR is most active, and 92% of the studies in one review of generative AI in health economic evaluation were conference abstracts,^33^ so our corpus understates operational activity.

Our classifier is a term dictionary, and its errors are large, measured, and reported rather than hidden. Precision against blinded independent classification was 51.3% overall and 29.6% for economic evaluation, so all domain counts should be read as upper bounds.

Our reference standard is itself a large language model rather than a human expert panel. This is a genuine circularity in an article about the reliability of these tools, and it cuts both ways: the blinded classifier may be systematically strict about what counts as HEOR, in which case our precision estimate is too low, or lenient on unfamiliar subfields, in which case some labels are understated. A human dual review of the same 300 records is the correct next step, and the sample and both label sets are archived so that anyone may perform it.^20^

Our denominator series contains an unresolved discrepancy of 273 records between the range query and the sum of per-year queries, and we restricted to English-language records, which biases the corpus toward North American and European practice.^89^

## CONCLUSIONS

The literature on AI in HEOR has grown by a factor of 29 since 2010 and reached 852 indexed records in 2025. Under blinded independent classification, approximately half of it is about something else, and the fastest growing label is the one that can least reliably be assigned. Of 2476 records, 38 were randomized trials and 17 were validation studies. No regulator or HTA body has yet been documented accepting or rejecting an AI-derived endpoint as such.

The measured gains are concentrated where ground truth is observable, which is to say in the clerical layer, and they are absent where the quantity of interest is a counterfactual. That division is stable across every workflow reviewed here and is the reason a scoping review of this field has to report its own error rate before it reports anything else. Part 2 asks what the regulatory and submission record says about the same division, and what reporting would make it auditable.

### Disclosures

The author reports no conflicts of interest related to this work. No funding was received for this study. The author is affiliated with an academic institution and a health economics consultancy that conducts real-world evidence studies for pharmaceutical and life sciences clients; no client had any role in the conception, conduct, analysis, or reporting of this review, and no client reviewed the manuscript before submission. The large language model classifier described in the Methods is a measured object of study in this review rather than an assistive writing tool. Its prompts, inputs, and outputs are archived in the replication package. No AI tool is listed as an author.

## Supporting information

Online Supplementary Material

## Data Availability

All search strings, retrieved records, the classification dictionary, the blinded classifier prompts and outputs, the analysis scripts, the figure generation code, and the complete reference library are available in the public replication package (https://github.com/obaser/ai-heor-scoping-review). No individual patient data were used.
