# Supplementary material for: Faster but Not Truer, Part 1: A Systematic Scoping Review of Artificial Intelligence in Health Economics and Outcomes Research
Source: J Health Econ Outcomes Res. 2026 Oct 15;13(2):148–60. doi: 10.36469/001c.171937 (PMC13676315; doi:10.36469/001c.171937)
Supplement: Supplementary file 1 [file jheor_2026_13_2_171937_371406.pdf]

Supplementary Appendix

Faster but Not Truer, Part 1: AI in Health Economics and Outcomes Research. *JHEOR*. 2026;13(2):148-160. doi:10.36469/jheor.2026.171937

Table S1 through Table S14 support the Methods and Results of Part 1. Every value is reproducible from the public replication package at https://github.com/obaser3791/ai-heor-scoping-review

Table S1. Complete PubMed Search Strings

Series	PubMed query string
all_pubmed	all[sb]
ai_all_medicine	("artificial intelligence"[tiab] OR "machine learning"[tiab] OR "deep learning"[tiab] OR "neural network"[tiab] OR "neural networks"[tiab] OR "large language model"[tiab] OR "large language models"[tiab] OR "natural language processing"[tiab] OR "generative AI"[tiab] OR "Artificial Intelligence"[Mesh])
heor_all	("health economics"[tiab] OR "outcomes research"[tiab] OR "pharmacoeconomics"[tiab] OR "cost-effectiveness"[tiab] OR "cost effectiveness"[tiab] OR "cost-utility"[tiab] OR "budget impact"[tiab] OR "health technology assessment"[tiab] OR "economic evaluation"[tiab] OR "quality-adjusted life"[tiab] OR "real-world evidence"[tiab] OR "real world evidence"[tiab] OR "Cost-Benefit Analysis"[Mesh] OR "Economics, Pharmaceutical"[Mesh] OR "Technology Assessment, Biomedical"[Mesh])
ai_heor	("artificial intelligence"[tiab] OR "machine learning"[tiab] OR "deep learning"[tiab] OR "neural network"[tiab] OR "neural networks"[tiab] OR "large language model"[tiab] OR "large language models"[tiab] OR "natural language processing"[tiab] OR "generative AI"[tiab] OR "Artificial Intelligence"[Mesh]) AND ("health economics"[tiab] OR "outcomes research"[tiab] OR "pharmacoeconomics"[tiab] OR "cost-effectiveness"[tiab] OR "cost effectiveness"[tiab] OR "cost-utility"[tiab] OR "budget impact"[tiab] OR "health technology assessment"[tiab] OR "economic evaluation"[tiab] OR "quality-adjusted life"[tiab] OR "real-world evidence"[tiab] OR "real world evidence"[tiab] OR "Cost-Benefit Analysis"[Mesh] OR "Economics, Pharmaceutical"[Mesh] OR "Technology Assessment, Biomedical"[Mesh])
ai_economic_evaluation	("artificial intelligence"[tiab] OR "machine learning"[tiab] OR "deep learning"[tiab] OR "neural network"[tiab] OR "neural networks"[tiab] OR "large language model"[tiab] OR "large language models"[tiab] OR "natural language processing"[tiab] OR "generative AI"[tiab] OR "Artificial Intelligence"[Mesh]) AND ("cost-effectiveness"[tiab] OR "cost effectiveness"[tiab] OR "cost-utility"[tiab] OR "economic evaluation"[tiab] OR "Cost-Benefit Analysis"[Mesh])
ai_hta	("artificial intelligence"[tiab] OR "machine learning"[tiab] OR "deep learning"[tiab] OR "neural network"[tiab] OR "neural networks"[tiab] OR "large language model"[tiab] OR "large language models"[tiab] OR "natural language processing"[tiab] OR "generative AI"[tiab] OR "Artificial Intelligence"[Mesh]) AND ("health technology assessment"[tiab] OR "Technology Assessment, Biomedical"[Mesh] OR "reimbursement"[tiab] OR "payer"[tiab] OR "market access"[tiab])
ai_rwe	("artificial intelligence"[tiab] OR "machine learning"[tiab] OR "deep learning"[tiab] OR "neural network"[tiab] OR "neural networks"[tiab] OR "large language model"[tiab] OR "large language models"[tiab] OR "natural language processing"[tiab] OR "generative AI"[tiab] OR "Artificial Intelligence"[Mesh]) AND ("real-world evidence"[tiab] OR "real world evidence"[tiab] OR "real-world data"[tiab] OR "claims data"[tiab] OR "administrative claims"[tiab] OR "electronic health record"[tiab] OR "Electronic Health Records"[Mesh])
ai_evidence_synthesis	("artificial intelligence"[tiab] OR "machine learning"[tiab] OR "deep learning"[tiab] OR "neural network"[tiab] OR "neural networks"[tiab] OR "large language model"[tiab] OR "large language models"[tiab] OR "natural language processing"[tiab] OR "generative AI"[tiab] OR "Artificial Intelligence"[Mesh]) AND ("systematic review"[tiab] OR "evidence synthesis"[tiab] OR "meta-analysis"[tiab] OR "literature screening"[tiab] OR "abstract screening"[tiab] OR "data extraction"[tiab])

Series	PubMed query string
ai_causal_inference	("artificial intelligence"[tiab] OR "machine learning"[tiab] OR "deep learning"[tiab] OR "neural network"[tiab] OR "neural networks"[tiab] OR "large language model"[tiab] OR "large language models"[tiab] OR "natural language processing"[tiab] OR "generative AI"[tiab] OR "Artificial Intelligence"[Mesh]) AND ("causal inference"[tiab] OR "propensity score"[tiab] OR "confounding"[tiab] OR "target trial"[tiab] OR "treatment effect"[tiab] OR "causal forest"[tiab] OR "targeted maximum likelihood"[tiab] OR "double machine learning"[tiab])
ai_decision_modeling	("artificial intelligence"[tiab] OR "machine learning"[tiab] OR "deep learning"[tiab] OR "neural network"[tiab] OR "neural networks"[tiab] OR "large language model"[tiab] OR "large language models"[tiab] OR "natural language processing"[tiab] OR "generative AI"[tiab] OR "Artificial Intelligence"[Mesh]) AND ("microsimulation"[tiab] OR "decision model"[tiab] OR "Markov model"[tiab] OR "discrete event simulation"[tiab] OR "decision-analytic"[tiab] OR "simulation model"[tiab])
ai_patient_reported	("artificial intelligence"[tiab] OR "machine learning"[tiab] OR "deep learning"[tiab] OR "neural network"[tiab] OR "neural networks"[tiab] OR "large language model"[tiab] OR "large language models"[tiab] OR "natural language processing"[tiab] OR "generative AI"[tiab] OR "Artificial Intelligence"[Mesh]) AND ("patient-reported outcome"[tiab] OR "patient reported outcome"[tiab] OR "health-related quality of life"[tiab] OR "utility elicitation"[tiab] OR "EQ-5D"[tiab])
ai_reporting_governance	("artificial intelligence"[tiab] OR "machine learning"[tiab] OR "deep learning"[tiab] OR "neural network"[tiab] OR "neural networks"[tiab] OR "large language model"[tiab] OR "large language models"[tiab] OR "natural language processing"[tiab] OR "generative AI"[tiab] OR "Artificial Intelligence"[Mesh]) AND ("reporting guideline"[tiab] OR "reporting standard"[tiab] OR "transparency"[tiab] OR "reproducibility"[tiab] OR "governance"[tiab] OR "algorithmic bias"[tiab] OR "risk of bias"[tiab])
llm_heor	("large language model"[tiab] OR "large language models"[tiab] OR "GPT-4"[tiab] OR "GPT-3"[tiab] OR "ChatGPT"[tiab] OR "generative AI"[tiab] OR "generative artificial intelligence"[tiab]) AND ("health economics"[tiab] OR "outcomes research"[tiab] OR "pharmacoeconomics"[tiab] OR "cost-effectiveness"[tiab] OR "cost effectiveness"[tiab] OR "cost-utility"[tiab] OR "budget impact"[tiab] OR "health technology assessment"[tiab] OR "economic evaluation"[tiab] OR "quality-adjusted life"[tiab] OR "real-world evidence"[tiab] OR "real world evidence"[tiab] OR "Cost-Benefit Analysis"[Mesh] OR "Economics, Pharmaceutical"[Mesh] OR "Technology Assessment, Biomedical"[Mesh])

All searches were executed on 9 September 2026 through the PubMed E-utilities interface. The primary review search is the ai_heor string restricted to publication years 2010 through 2025. The remaining strings are the denominator series reported in Table S5.

Table S2. Complete Classification Dictionary

Label	Label type	Key	Terms and patterns
Economic evaluation	Application domain	economic_evaluation	cost[-]effectiveness; cost[-]utility; cost[-]benefit; incremental cost; \bICER\b; quality[-]adjusted life; \bQALY; budget impact; economic evaluation; cost[-]consequence; willingness[-]to[-]pay threshold; net monetary benefit; health economic model
Decision modeling and simulation	Application domain	decision_modeling	microsimulation; markov model; discrete event simulation; decision[-]analytic; decision tree model; state[-]transition model; simulation model; agent[-]based model; metamodel; emulator; probabilistic sensitivity analysis; value of information
Evidence synthesis	Application domain	evidence_synthesis	systematic review; scoping review; meta[-]analys; evidence synthesis; literature screening; abstract screening; title and abstract; data extraction; rapid review; umbrella review; living review; network meta
Real-world data and causal inference	Application domain	rwd_causal	real[-]world evidence; real[-]world data; claims data; administrative claims; administrative data; electronic health record; \bEHR\b; registry data; causal inference; propensity score; confound; target trial; instrumental variable; difference[-]in[-]difference; treatment effect heterogeneity; causal forest; targeted maximum likelihood; double machine learning; external control arm; synthetic control
Outcome and cost prediction	Application domain	outcome_prediction	prognostic model; prediction model; predictive model; risk prediction; risk stratification; readmission; mortality prediction; predicting (?:cost|expenditure|utilization|length of stay); length of stay; resource utilization prediction; clinical deterioration; \bAUC\b; \bAUROC\b; c[-]statistic; discrimination and calibration
Patient-reported outcomes and preferences	Application domain	pro_preferences	patient[-]reported outcome; \bPROM\b; \bPROs\b; health[-]related quality of life; \bHRQoL\b; \bEQ[-]5D\b; \bSF[-]36\b; utility (?:value|elicitation|score); discrete choice experiment; stated preference; conjoint analysis; patient preference
HTA, payer, and market access	Application domain	hta_access	health technology assessment; \bHTA\b; reimburse; market access; formulary; payer; coverage decision; pricing; value dossier; \bNICE\b; \bIQWiG\b; \bCADTH\b; \bICER (?:report|review); joint clinical assessment; managed entry agreement; value[-]based

<table>
<tr><th>Label</th><th>Label type</th><th>Key</th><th>Terms and patterns</th></tr>
<tr><td>Governance, reporting, and validity</td><td>Application domain</td><td>governance_reporting</td><td>reporting guideline; reporting standard; transparen; reproducib; replicab; algorithmic bias; algorithmic fairness; risk of bias; \bTRIPOD\b; \bPROBAST\b; \bCHEERS\b; \bCONSORT\b; \bSPIRIT\b; \bDECIDE[-]AI\b; \bPRISMA\b; regulatory (?:approval|submission|guidance|acceptance); governance; ethic; explainab; interpretab; accountab; hallucinat; fabricat</td></tr>
<tr><td>Generative AI and large language models</td><td>Method category (evaluated in order of specificity)</td><td>generative_llm</td><td>large language model; \bLLM\b; \bLLMs\b; \bGPT-?[345]\b; ChatGPT; generative (?:AI|artificial intelligence|pre[-]?trained); \bBERT\b; transformer; foundation model; Claude; Gemini; \bLlama\b; prompt engineering; retrieval[-]augmented; \bGPT\b</td></tr>
<tr><td>Natural language processing (pre-generative)</td><td>Method category (evaluated in order of specificity)</td><td>nlp</td><td>natural language processing; \bNLP\b; text mining; named entity recognition; clinical notes; unstructured text; free[-]text; topic model; word embedding</td></tr>
<tr><td>Deep learning</td><td>Method category (evaluated in order of specificity)</td><td>deep_learning</td><td>deep learning; neural network; convolutional; recurrent neural; \bCNN\b; \bLSTM\b; deep neural; autoencoder; generative adversarial; \bGAN\b</td></tr>
<tr><td>Classical machine learning</td><td>Method category (evaluated in order of specificity)</td><td>classical_ml</td><td>machine learning; random forest; gradient boosting; \bXGBoost\b; support vector; \bLASSO\b; elastic net; decision tree; \bk[-]means\b; clustering; ensemble learning; supervised learning; unsupervised learning; reinforcement learning</td></tr>
<tr><td>Artificial intelligence, unspecified</td><td>Method category (evaluated in order of specificity)</td><td>ai_general</td><td>artificial intelligence; \bAI\b</td></tr>
</table>

Terms are regular expressions applied case insensitively to the concatenated title, abstract, and MeSH terms. Domain labels are non-exclusive. Method categories are evaluated in the order shown and the first match is assigned, so that generative AI takes precedence over classical machine learning and unspecified AI is assigned only when no more specific term is present. The measured error rates of this dictionary are reported in Table 2 and Table S8.

Table S3. PRISMA-ScR Flow with Itemized Exclusion Reasons

Stage or reason	Records (n)
**Records identified in PubMed**	**2642**
**Duplicate records removed**	**14**
**Records screened**	**2628**
**Records excluded, total**	**152**
no abstract available for classification	70
not published in English	38
excluded publication type (Editorial)	19
excluded publication type (Preprint)	10
excluded publication type (Letter)	7
outside publication window 2010-2025	4
excluded publication type (Retracted Publication)	4
**Records included**	**2476**
**Precision-adjusted included records (point estimate)**	**1271**
**Precision-adjusted included records (95% CI)**	**1131 to 1410**

Exclusions were applied programmatically in the order listed and each record is counted once, under the first applicable reason. The precision-adjusted estimate applies the search precision measured in Table 2 to the included count and is reported for interpretation, not as a screening result.

Table S4. Included Records per Year by Application Domain and Method Category, 2010-2025

Year	Total	Econ eval	RWD causal	Governance	Prediction	Evidence synth	HTA access	Decision model	PRO pref	No domain	AI unspec	Classical ML	Deep learn	No method	Generative	NLP
2010	29	9	0	5	2	1	4	1	0	24	0	4	1	24	0	0
2011	28	16	0	1	2	3	2	0	1	25	0	2	1	25	0	0
2012	47	23	3	3	8	5	1	2	1	37	0	4	4	37	0	2
2013	31	20	1	4	2	8	2	0	1	29	0	0	1	29	0	1
2014	34	21	2	0	3	5	3	1	1	31	0	1	2	31	0	0
2015	24	6	3	1	4	0	2	0	0	12	1	8	1	12	0	2
2016	23	13	3	2	2	5	1	1	2	13	0	5	3	13	0	2
2017	36	16	4	2	6	4	1	0	1	14	2	10	9	14	0	1
2018	48	25	11	6	7	3	2	3	1	12	10	17	6	12	0	3
2019	91	37	22	11	19	8	7	2	2	26	12	26	17	26	0	10
2020	130	63	25	15	22	9	7	7	2	20	20	43	36	20	0	11
2021	163	90	42	28	23	17	17	8	5	17	46	47	36	17	1	16
2022	232	151	47	39	47	28	28	18	8	43	56	67	52	43	4	10
2023	270	173	62	58	43	25	25	18	11	42	75	73	44	42	13	23
2024	438	308	93	82	72	41	41	22	7	36	133	118	89	36	38	24
2025	852	588	178	237	131	121	66	32	33	64	327	190	143	64	103	25

Domain columns are non-exclusive and do not sum to the included total. Method columns are exclusive and sum to the included total. Column abbreviations, in order: Econ eval, economic evaluation; RWD causal, real-world data and causal inference; Governance, governance, reporting, and validity; Prediction, outcome and cost prediction; Evidence synth, evidence synthesis; HTA access, HTA, payer, and market access; Decision model, decision modeling and simulation; PRO pref, patient-reported outcomes and preferences; No domain, records to which the dictionary assigned no domain label; AI unspec, artificial intelligence unspecified; Classical ML, classical machine learning; Deep learn, deep learning; No method, no AI method term identified; Generative, generative AI and large language models; NLP, natural language processing before the generative era.

Table S5. Annual PubMed Denominator Series, 2010-2025

Series	2010	2011	2012	2013	2014	2015	2016	2017	2018	2019	2020	2021	2022	2023	2024	2025
ai_all_medicine	5540	6462	6896	8451	8585	8908	9712	11891	16558	23575	33021	46324	58612	65233	80590	110494
ai_causal_inference	14	20	25	41	40	45	79	72	108	128	198	322	474	492	614	977
ai_decision_modeling	24	24	20	39	33	43	51	50	59	92	80	114	167	157	164	194
ai_economic_evaluation	21	29	47	41	45	25	25	31	40	65	104	119	191	223	376	685
ai_evidence_synthesis	41	49	89	110	101	93	116	154	202	329	487	867	1221	1511	2023	3509
ai_heor	39	39	56	51	49	38	31	46	61	116	167	190	275	315	511	945
ai_hta	24	16	21	15	11	13	10	17	20	29	40	64	82	72	108	208
ai_patient_reported	3	10	12	5	10	3	7	11	15	31	53	77	96	109	119	195
ai_reporting_governance	41	49	67	66	58	74	76	104	194	321	513	788	1087	1376	2243	5136
ai_rwe	66	89	125	204	214	302	226	309	418	654	713	917	951	1097	1648	2398
all_pubmed	942369	1020845	1090505	1150126	1205918	1258163	1285527	1304378	1351710	1418998	1641241	1787582	1783561	1697844	1739776	1881997
heor_all	5083	5307	5918	6534	7730	7946	8196	8691	8699	9244	9958	10591	10522	10323	12265	15641
llm_heor	0	0	0	0	0	0	0	0	0	0	0	0	0	6	34	105

All series retrieved 9 September 2026. Query strings are given in Table S1. The range form of the ai_heor query returned 2,656 records where the sum of the sixteen per year queries was 2,929, a difference of 273 records. The most likely explanation is records with a MedlineDate spanning two calendar years, which are counted in both per year queries. The per year series was used for trend estimation and the range query for the PRISMA flow.

Table S6. Forty Most Frequent Publication Venues Among Included Records

Journal	Included records (n)	% of included
Scientific Reports	79	3.19
Sensors (Basel, Switzerland)	60	2.42
Cureus	43	1.74
Journal of Medical Internet Research	41	1.66
BMJ Open	36	1.45
PloS one	35	1.41
Value in Health: the Journal of the International Society for Pharmacoeconomics and Outcomes Research	32	1.29
Journal of Clinical Medicine	27	1.09
NPJ Digital Medicine	26	1.05
Frontiers in Public Health	20	0.81
Studies in Health Technology and Informatics	19	0.77
Diagnostics (Basel, Switzerland)	19	0.77
Frontiers in Pharmacology	19	0.77
JMIR Medical Informatics	18	0.73
Cancers	17	0.69
BMC Medical Informatics and Decision Making	16	0.65
Journal of the American Medical Informatics Association : JAMIA	16	0.65
Digital Health	15	0.61
Heliyon	15	0.61
European Radiology	14	0.57
JMIR Research Protocols	14	0.57
Computers In Biology and Medicine	14	0.57
Journal of Robotic Surgery	13	0.53
International Journal of Technology Assessment In Health Care	13	0.53
Trials	12	0.48
International Journal of Environmental Research and Public Health	12	0.48
Medical Decision Making: An International Journal of the Society for Medical Decision Making	11	0.44

Journal	Included records (n)	% of included
Surgical Endoscopy	11	0.44
Journal of Comparative Effectiveness Research	10	0.40
International Journal of Medical Informatics	10	0.40
Journal of Environmental Management	10	0.40
Artificial Intelligence In Medicine	10	0.40
Journal of Medical Economics	10	0.40
Frontiers in Artificial Intelligence	9	0.36
Health Technology Assessment (Winchester, England)	9	0.36
BJU International	9	0.36
Expert Review of Medical Devices	8	0.32
JCO Clinical Cancer Informatics	8	0.32
Journal of Biomedical Informatics	8	0.32
Healthcare (Basel, Switzerland)	8	0.32

Journal names are as indexed in PubMed. The 2,476 included records appeared in more than 900 distinct venues, so the concentration shown here is low by design.

Table S7. Indexed Publication Types Among Included Records

Publication type	Included records (n)	% of included
Journal Article	2,471	99.80
Review	674	27.22
Research Support, Non-U.S. Gov't	492	19.87
Systematic Review	121	4.89
Research Support, N.I.H., Extramural	105	4.24
Comparative Study	91	3.68
Multicenter Study	54	2.18
Scoping Review	42	1.70
Randomized Controlled Trial	38	1.53
Research Support, U.S. Gov't, Non-P.H.S.	37	1.49
Clinical Trial Protocol	31	1.25
Meta-Analysis	27	1.09
Validation Study	17	0.69
Observational Study	17	0.69
Evaluation Study	14	0.57
Consensus Statement	12	0.48
Research Support, U.S. Gov't, P.H.S.	11	0.44
Case Reports	7	0.28
Clinical Trial	6	0.24
Conference Proceedings	4	0.16
Video-Audio Media	3	0.12
Comment	3	0.12
Research Support, N.I.H., Intramural	3	0.12
Pragmatic Clinical Trial	3	0.12
Equivalence Trial	2	0.08
Practice Guideline	2	0.08
Adaptive Clinical Trial	2	0.08

Publication type	Included records (n)	% of included
Controlled Clinical Trial	2	0.08
Historical Article	1	0.04
Clinical Trial, Phase IV	1	0.04
Dataset	1	0.04
Randomized Controlled Trial, Veterinary	1	0.04
Introductory Journal Article	1	0.04

Publication types are as indexed in PubMed and a record may carry more than one, so counts sum to more than 2,476. Randomized controlled trials account for 38 records (1.5%) and validation studies for 17 (0.7%).

Table S8. Full Agreement Statistics for the Validation Analysis

Label	Dictionary (n)	Blinded classifier (n)	Observed agreement (%)	Cohen kappa	PABAK	Precision (%)	Recall (%)	F1 (%)
Economic evaluation	186	69	51.7	0.144	0.033	29.6	79.7	43.1
Decision modeling and simulation	16	30	94.0	0.579	0.880	87.5	46.7	60.9
Evidence synthesis	33	46	92.3	0.666	0.847	84.8	60.9	70.9
Real-world data and causal inference	64	75	90.3	0.729	0.807	85.9	73.3	79.1
Outcome and cost prediction	43	50	85.7	0.453	0.713	58.1	50.0	53.8
Patient-reported outcomes and preferences	8	22	93.3	0.306	0.867	62.5	22.7	33.3
HTA, payer, and market access	21	25	94.0	0.576	0.880	66.7	56.0	60.9
Governance, reporting, and validity	65	68	84.3	0.546	0.687	66.2	63.2	64.7
Method category, 6-category multiclass	300	300	73.7	0.667				
Generative AI or LLM flag			97.0	0.674	0.940			
Mean domain kappa (median observed agreement)			92.3	0.500				

Paired records: 300. Cohen kappa below 0.20 indicates slight agreement, 0.21 to 0.40 fair, 0.41 to 0.60 moderate, 0.61 to 0.80 substantial. The final row reports the mean domain kappa and the median observed agreement across the eight domains.

Table S9. Search Precision, Overall and Stratified by Dictionary Domain Assignment

Stratum	n sampled	n judged HEOR relevant	Precision (%)	95% CI lower	95% CI upper
All included records	300	154	51.3	45.7	56.9
Dictionary assigned ≥1 HEOR domain	271	142	52.4	46.5	58.3
Dictionary assigned no HEOR domain	29	12	41.4	25.5	59.3

Precision is the proportion of sampled records judged genuinely relevant to health economics and outcomes research by the blinded independent classifier. Confidence intervals are exact binomial (Clopper-Pearson). The stratification shows that the dictionary carries signal, since precision is higher among records to which it assigned at least one domain, but not enough of it.

Table S10. Primary Studies of Large Language Model Performance in Study Screening

Study focus	Records screened	Model	Sensitivity	Specificity or agreement	Reported efficiency	Provenance
Two systematic review data sets	1,381 and 3,146	GPT-4, adjusted prompting	0.962	0.996	Approximately 110 records per minute at US$59	Peer reviewed
Multiple reviews	22,665 citations	Multiple LLMs	0.811 to 0.965	0.258 to 0.804	10,279 false positives in the worst configuration	Peer reviewed
Multiple reviews	16,669 citations	GPT-4	0.85	0.98, with a median specificity gap of 0.48 across reviews	Not reported	Peer reviewed
Human versus LLM screening	Not applicable	GPT-4	0.42	Kappa 0.34 vs human kappa 0.77	Not reported	Peer reviewed
Environmental and health evidence	11,984 records	LLM prioritisation	Recall above 95% target	Work saved over sampling 0.55	Approximately 75% of screening effort saved	Peer reviewed
Agentic HEOR literature review	Not stated	Multi-agent, human in the loop	F1 0.917 to 0.977	Kappa 0.844 to 0.906 vs human	Not reported separately	Peer reviewed
Time and motion, hybrid vs autonomous	234 publications	Not stated	Hybrid recall 80.6%; autonomous recall 48.4%; unaided human recall 88.7%	Hybrid accuracy 97.0%; unaided human accuracy 90.1%	Hybrid 51.4% time saved; autonomous 81.1% time saved	Conference abstract

Sensitivity is the proportion of truly eligible records retained. Specificity is the proportion of truly ineligible records correctly excluded. Where a range is given, it spans models or reviews within the same study. The consistent pattern is acceptable sensitivity with unstable specificity, and between-review variance exceeding between-model variance.

Table S11. Primary Studies of Large Language Model Performance in Data Extraction and Downstream Analysis

Task	Scale	Model	Result	Human comparator	Provenance
Extraction of 160 predefined items	Not stated	Claude 2	96.3% accuracy	Human extraction	Peer reviewed
Randomized trial extraction	Not stated	Not stated	94.8% accuracy at 88 seconds per trial	Human extraction	Peer reviewed
Extraction across 107 randomized trials	12,814 extractions	Claude 3.5	96.2% accuracy; time 86.9 to 14.7 minutes	Human 95.3%; human assisted by model 97.9%	Peer reviewed
Automated cost effectiveness model coding in R	30 scripts	GPT-4	14 of 15 error free in the lung cancer set; every error free script within 1% of the published ICER	Published model results	Peer reviewed
Network meta-analysis input extraction	Not stated	GPT-4	Greater than 99% extraction accuracy	Human extraction	Peer reviewed
Expected value of partial perfect information	12.5 million model runs	Metamodel	More than 1,000 days reduced to approximately 2 days	Brute force nested simulation	Peer reviewed
Reference generation, a cautionary comparison	471 references	GPT-3.5, GPT-4, Bard	Hallucination 39.6%, 28.6%, 91.4%; DOI correct in 16% and 20%	Verified bibliographic records	Peer reviewed
Reference generation, a cautionary comparison	636 citations	GPT-3.5, GPT-4	55% and 18% fabricated, rising to 70% for book chapters	Verified bibliographic records	Peer reviewed

Where both a model and a human arm are reported, the comparison of interest is the combination arm rather than either alone.

Table S12. Machine Learning Methods for Causal Inference and Their Translation into HEOR

Method or study	Contribution	Key quantitative finding
High dimensional propensity score	Algorithmic covariate selection from claims	Relative risk moved from 1.09 crude to 0.94 with 15 investigator covariates to 0.88 with 500 selected covariates in 49,653 users; a conventional estimate of 0.84 (0.56 to 1.26) became 0.62 (0.43 to 0.91)
Performance under unmeasured confounding	Empirical assessment of hdPS robustness	Characterises the conditions under which the approach reduces or fails to reduce bias
Targeted maximum likelihood estimation	Doubly robust semiparametric estimation	Foundational method, with pharmacoepidemiologic translation
Super learning	Ensemble selection by cross validation	Foundational method underlying doubly robust implementations
Causal forests	Heterogeneous treatment effect estimation with valid inference	Foundational method; applied to heterogeneity in cost effectiveness analysis
Double or debiased machine learning	Orthogonalised estimation of treatment effects	Foundational method for high dimensional nuisance parameters
hdPS weighting failure mode	Simulation of estimator behaviour	Bias 0.094 with 23.6% confidence interval coverage, against 0.018 and 91.4% for a modified procedure
Trial emulation, PARADIGM-HF	Comparison of propensity model classes against a trial benchmark	AUC 0.79 gave HR 0.93; AUC 0.95 gave HR 0.61; trial HR 0.81 (0.61 to 1.06)
Heterogeneous treatment effects with real-world data	Scoping review	1,743 records screened, 74 studies included
Transportability and generalizability	Methods for moving estimates between populations	Reviewed methods for target population extrapolation in HEOR contexts
Target trial framework	Design discipline for real-world evidence	Recommended as the organizing framework for observational comparative effectiveness
PALISADE checklist	Credibility instrument for real-world data studies	Written for precisely the failure modes above and referenced by NICE

The recurring finding is that predictive performance and evidentiary performance are separable. Optimizing discrimination in a propensity model can degrade the covariate balance that identification requires.

Table S13. Natural Language Processing Performance for HEOR-Relevant Variable Extraction from Unstructured Data

Variable or task	Scale	Reported performance	Reference standard	Provenance
Social and behavioral determinants	Not stated	Recorded prevalence rose from 0.03% to 8.3% in claims to 1.40% to 36.3% with notes; walking difficulty 10.7% to 38.0%; lack of social support text only in 99.8% of cases	Structured claims	Peer reviewed
Adverse social determinants in oncology	48 patients with documented determinants	44 of 48 (91.7%) had no corresponding Z code; fewer than 2% of inpatient hospitalizations carried any Z coded diagnosis	Structured coding	Peer reviewed
ECOG performance status	79,698 patients, 495,862 notes	AUROC 0.95; imputed poor performance status HR 11.9	Documented performance status	Peer reviewed
Performance status, Veterans Affairs implementation	Not stated	98.5% accuracy	Manual review	Peer reviewed
PD-L1 status	Not stated	Micro F1 0.925	Manual abstraction	Peer reviewed
Oncology staging and histology	200 patients drawn from more than 27 million	Lung neoplasm 100% precision and 100% recall; NSCLC histology 99% and 88%; histology linked to neoplasm 98% and 79%; stage value 98.8% and 92%; metastasis site 97% and 89%	Human abstraction	Peer reviewed
Cancer registry TNM staging	Not stated	T stage F1 0.89; M stage F1 0.24	Registry abstraction	Peer reviewed
Real-world progression free survival	316 metastatic breast cancer patients	Sentence level 98.2%; patient level within 30 days 88%; median rwPFS 20 months (18 to 25) computational, 22 months (15 to 35) in the validation subset, 25 months (15 to 35) manual; 30 months from radiology reports vs 23 months from clinical notes	Manual curation of 100 patients	Peer reviewed
Human abstracted real-world response, reference standard	3,248 patients, 85.4% with at least one radiographic assessment	Inter-abstractor agreement 0.94 (0.92 to 0.96) and 0.82 (0.78 to 0.86); 15.0 minutes per patient line (IQR 7.8 to 28.1); Spearman 0.99 vs trial ORR	Second human abstractor	Peer reviewed
Scaled machine learning real-world response	Test subset of 4,047	r = 0.86 against human abstraction	Human abstraction	Conference abstract
Cross tumor LLM extraction	14 cancer types	F1 similar to expert human abstractors; numerical values not disclosed	Expert abstraction	Conference abstract

Variable or task	Scale	Reported performance	Reference standard	Provenance
AI augmented real-world data	Not stated	Recall 49.9% to 93.2%; precision 73.6% to 93.5%; completeness 46.1% (38.2 to 54.0) to 96.6% (85.8 to 107.4)	Traditional structured approach	Peer reviewed
Vendor accuracy claim	Not stated	Greater than 92% accuracy	Not stated	Company communicatio n
AI derived HEOR variables, second vendor	Not applicable	No published validation located	Not applicable	Not available

Approximately 80% of clinical data is unstructured. The pattern across studies is that the variable a cohort definition or an endpoint depends on is frequently the variable extracted worst, which is why item 5 of TRACE-HEOR requires disaggregated reporting.

Table S14. Synthetic Data, Digital Twins, and Model-Based External Control Arms

Study or instrument	Setting	Key quantitative finding
Generator comparison for control arm simulation	Oncology control arms	CART based generation recovered the real median progression free survival inside the 95% CI in 88.8% to 98.1% of replicates, against 6.5% to 37.8% for a conditional tabular GAN
Synthetic control arm in myelodysplastic syndromes	7,133 patients	Clinical fidelity 93%, genomic fidelity 90%; 187 luspatercept matched synthetic controls
Synthetic data as external controls in scarce single arm trials	Rare disease	Evaluates feasibility and the conditions for acceptable inference
AI generated digital twins	6,736 subjects in Alzheimer disease	6,236 of 6,728 subjects with recorded race were White and 152 were Black, which bounds what the synthetic population can be used to claim
Training on synthetic electronic health records	Not stated	Model trained on synthetic records performed 0.026 lower in AUC than one trained on real records
Reporting practices in synthetic health data	73 studies	70 (95%) evaluated utility; 31 of 67 (46%) claiming a privacy purpose evaluated privacy; 8 of 73 (11%) used a differentially private generator
FDA approvals accepting external control data	45 approvals, 2000 to 2019	36 of 45 (80%) in rare disease; none used prospectively collected natural history controls
Statistical limits of external controls for regulatory use	Methodological analysis	Characterises the bias and inference constraints for regulatory acceptance
EMA qualification opinion on prognostic covariate adjustment	Regulatory opinion	Qualified, while stating that excluding dropout data would not be acceptable for regulatory purposes

The regulatory record on external controls is more restrictive than the synthetic data literature acknowledges, and generator choice dominates whether the target quantity is recovered at all.
